# Preparation of Non-Covalent BPTCD/g-C_3_N_4_ Heterojunction Photocatalysts and Photodegradation of Organic Dyes Under Solar Irradiation

**DOI:** 10.3390/nano15141131

**Published:** 2025-07-21

**Authors:** Xing Wei, Gaopeng Jia, Ru Chen, Yalong Zhang

**Affiliations:** 1 Faculty of Environmental Science and Engineering, Yancheng Institute of Technology, Yancheng 224051, China; 15396805562@163.com; 2 Institute of Textile and Apparel, Yancheng Institute of Technology, Yancheng 224051, China; cr1986634019@163.com (R.C.); 18662029598@189.cn (Y.Z.)

**Keywords:** heterojunction photocatalyst, g-C_3_N_4_, 3,3′,4,4′-benzophenone tetracarboxylic dianhydride, organic pollutants

## Abstract

In this study, the BPTCD/g-C_3_N_4_ heterojunction photocatalyst was successfully prepared by the hydrothermal method. BPTCD (3,3′,4,4′-benzophenone tetracarboxylic dianhydride) is immobilised on the surface of g-C_3_N_4_ by non-covalent π-π stacking. The BPTCD/g-C_3_N_4_ heterojunction photocatalyst is an all-organic photocatalyst with significantly improved photocatalytic performance compared with g-C_3_N_4_. BPTCD/g-C_3_N_4_-60% was able to effectively degrade MO solution (10 mg/L) to 99.9% and 82.8% in 60 min under full spectrum and visible light. The TOC measurement results indicate that MO can ultimately be decomposed into H_2_O and CO_2_ through photocatalytic action. The photodegradation of methyl orange by BPTCD/g-C_3_N_4_ composite materials under sunlight is mainly attributed to the successful construction of the heterojunction structure and its excellent π-π stacking effect. Superoxide radicals (^•^O_2_^−^) were found to be the main active species, while ^•^OH and h^+^ played a secondary role. The synthesised BPTCD/g-C_3_N_4_ also showed excellent stability in the activity of photodegradation of MO in wastewater, with the performance remaining above 90% after three cycles. The mechanism of the photocatalytic removal of MO dyes was also investigated by the trap agent experiments. Additionally, BPTCD/g-C_3_N_4_-60% demonstrated exceptional photodegradation performance in the degradation of methylene blue (MB). BPTCD/g-C_3_N_4_ heterojunctions have great potential to degrade organic pollutants in wastewater under solar irradiation conditions.

## 1. Introduction

With the rapid economic development and industrialisation, environmental pollution has become an important challenge for all countries [1]. Organic dyes are one of the major pollutants in wastewater treatment [2]. These organic dyes are widely used in various industries such as textiles, plastics, leather, paints, and paper [3]. A large amount of dyes are discharged into water bodies as polluted wastewater every year. Due to their water-soluble nature, organic dyes are highly migratory in water bodies [4]. In addition, they are highly toxic, posing a serious threat to human health and the ecological environment, and may even cause serious consequences such as teratogenicity and carcinogenicity [5,6].

In recent years, organic semiconductor photocatalysts, which possess outstanding optoelectronic properties, have attracted significant attention from researchers and have been successfully applied in the field of photocatalysis [7]. The advantages of organic semiconductors over inorganic semiconductors are the designability of their molecular structure, broad spectral absorption, low preparation cost, environmental friendliness, and functional diversity [8,9,10]. g-C_3_N_4_ (graphite carbon nitride) is a two-dimensional structure composed of sp^2^ hybrid carbon (C) and nitrogen (N) atoms and has attracted extensive attention from researchers due to its physical and chemical properties, such as a narrow band gap, certain conductivity, excellent light absorption performance, and good chemical stability [11]. Since the first report of the use of g-C_3_N_4_ for photocatalytic water decomposition in 2009, this organic polymer semiconductor has been used in a wide range of applications such as photolysis of water, fuel cells, organic synthesis, and pollutant degradation [12,13,14,15]. However, its low utilisation of visible light, fast photogenerated carrier recombination, and low charge separation efficiency limit the wide application of this catalyst [16,17,18]. To address the intrinsic limitations of g-C_3_N_4_ and boost its photocatalytic activity, researchers have employed a range of strategies for its modification, including doping, morphological tuning, and surface engineering [19,20,21].

BPTCD (3,3′,4,4′-benzophenone tetracarboxylic dianhydride) consists of two benzene rings connected by a ketone group (C=O), each with two anhydride groups to form a tetraacidic dianhydride structure. It is a derivative of benzophenone and exhibits excellent thermal, chemical, and reactive properties [22]. BPTCD is sensitive to UV light and has no photocatalytic activity in visible light. In addition, the molecular structure of BPTCD contains conjugated units, such as benzene rings and carbonyl groups, and thus has a π-electron conjugation system. BPTCD is extensively utilised in the polyimides, polymer films, and synthesis of photosensitive materials [23,24,25]. Wang et al. discovered that BPTCD could be condensed and polymerised with 4,4′,4″-triaminotriphenylamine (TPA) to form polyimide (PPI) under mild conditions, which exhibited good photocatalytic activity for MB under visible light [26]. Yi et al. utilised BPTCD to construct nanophotoactive film materials that demonstrated good photocatalytic properties for reactive red 195 under ultraviolet irradiation [27]. In addition, the anhydride functional group can react with the amino group of g-C_3_N_4_, and g-C_3_N_4_-based photocatalysts can be prepared by using the imide bonding method [28]. However, this covalent functionalisation method destroys the π conjugation system of g-C_3_N_4_. Compared with covalent functionalisation, non-covalent functionalisation does not significantly alter the π-conjugation system of g-C_3_N_4_, thereby preserving the high conductivity of its electrons [29,30].

Although BPTCD is widely used in polyimide synthesis and other fields, the investigation into its morphological features and photoelectric properties is like a blank slate, and preparative studies on its use as a composite photocatalyst for the degradation of organic pollutants are even less frequently reported. In this paper, the morphology of BPTCD and its photoelectric properties were characterised in detail, and the BPTCD/g-C_3_N_4_ heterojunction photocatalysts consisting of BPTCD and g-C_3_N_4_ were successfully prepared by a one-step hydrothermal method. The physicochemical and optical properties of the obtained materials were thoroughly analysed and characterised. The photocatalytic performance of BPTCD/g-C_3_N_4_ heterojunction photocatalysts was systematically investigated and successfully applied to the photodegradation experiments of methyl orange (MO) and methylene blue (MB) under simulated sunlight. The cycling stability of the BPTCD/g-C_3_N_4_ heterojunction photocatalysts was discussed, and the radical species and potential degradation mechanisms of the BPTCD/g-C_3_N_4_ heterojunction photocatalysts were elaborated. The photocatalytic activity of different environmental factors was studied in detail to analyse its mechanism of action. It offers a novel and environmentally friendly method for effectively degrading dye wastewater, which holds significant environmental importance for improving dye wastewater pollution.

## 2. Materials and Methods

### 2.1. Materials

The chemical reagents used in the experiments included melamine (Melamine), isopropyl alcohol (IPA), anhydrous sodium sulphate (Na_2_SO_4_, 99%), benzoquinone (BQ, C_6_H_4_O_2_, 99%), and 3,3′,4,4′-benzophenone tetracarboxylic dianhydride (BPTCD, C_17_H_6_O_7_, CAS: 2421-28-5, ≥99%), which were purchased from Shanghai McLean Biochemistry Science and Technology Co., Ltd. (Shanghai, China). Methyl Orange (MO, AR grade) and Methylene Blue (MB, AR grade) were provided by Shanghai Shanpu Chemical Co. (Shanghai, China). Disodium ethylenediaminetetraacetic acid (EDTA-2Na) was obtained from Aladdin Chemical Reagent Co., Ltd. (Shanghai, China). All the above reagents were of analytical pure grade and used directly without purification. Deionised water (DI water) was used as the solvent for the preparation of all the solutions.

### 2.2. Photocatalysts Preparation

#### 2.2.1. Preparation of g-C_3_N_4_ Nanosheets

Preparation of g-C_3_N_4_ nanosheets by direct calcination pyrolysis of melamine. First, 15 g of melamine was placed in a crucible for pyrolysis. The melamine was then heated to 550 °C at a ramping rate of 5 °C/min and kept at this temperature for 3 h. The melamine was then heated to 550 °C at a ramping rate of 5 °C/min. After the muffle furnace was naturally cooled to room temperature, the resulting yellowish-coloured lumps of g-C_3_N_4_ were removed and collected by grinding them into powder form using a mortar and pestle. The collected powder was washed three times each with deionised water and anhydrous ethanol successively and finally dried in an oven at 60 °C.

#### 2.2.2. BPTCD/g-C_3_N_4_ Heterojunction Photocatalyst

Synthesis of BPTCD-functionalised g-C_3_N_4_ heterojunction photocatalysts using a typical hydrothermal-based approach. The total mass of g-C_3_N_4_ and BPTCD was kept as 800 mg. Different masses of g-C_3_N_4_ were placed in 100 mL of deionised water and ultrasonically stirred for 30 min, followed by the addition of different masses of BPTCD to the g-C_3_N_4_ suspension and continued stirring for 30 min for uniform dispersion. The dispersed mixture was transferred to a 150 mL hydrothermal synthesis reactor and kept at 160 °C for 12 h. After cooling to room temperature, the mixture was washed three times each with deionised water and anhydrous ethanol, respectively. Finally, the substance was washed and then placed in the oven at 60 °C for 8 h. A series of BPTCD/g-C_3_N_4_ samples were also prepared by controlling the ratio of raw materials, which were labelled BPTCD/g-C_3_N_4_-X (where X represents the amount of BPTCD fixed; X = 30%, 40%, 50%, 60%, 70%). The BPTCD/g-C_3_N_4_-60% photocatalyst, for example, is a composite of 0.32 g of g-C_3_N_4_ and 0.48 g of BPTCD.

### 2.3. Characterisation

The physicochemical properties of the materials were systematically resolved by multiscale characterisation techniques; field emission scanning electron microscopy (SEM, Nova Nano SEM 450, Milpitas, CA, USA) and transmission electron microscopy (TEM, JEM-1400 Plus, Tokyo, Japan) were used to analyse the surface morphology of the specimens and the microregion structure on the nanoscale, respectively. The crystal structure information (FTIR) was collected by X-ray polycrystalline diffractometer (X PERT3 POWDER, Almelo, The Netherlands); molecular vibration spectra were recorded by Fourier-transform infrared spectrometer (NEXUF-670, NICOLET, Mountain, WI, USA) in the 4000–400 cm^−1^ wave number interval to record molecular vibration characteristic spectra. X-ray photoelectron spectrometer (XPS, ESCALAB 250Xi, Shenzhen, China) was used to analyse the chemical state distribution of surface elements. For the evaluation of optical properties, a UV-visible diffuse reflectance spectroscopy system (UV-2450, Shimadzu, Kyoto, Japan) was used to determine the photoresponsive properties of the materials in the 200–800 nm band using barium sulphate as the reference substrate. Analysed specific surface area, pore size, and pore volume using the specific surface area test method (BTE, Kanta nova2000, Hickman, TN, USA). Use a total organic carbon analyser to test the degree of mineralisation in the solution (TOC, multi N/C UV HS, Hamburg, Germany). The characterisation of the photoelectrochemical behaviour was accomplished by a three-electrode system: ITO conductive glass as the working electrode, an Ag/AgCl electrode as the reference electrode, and a platinum sheet as the counter electrode in 0.5 mol/L sodium sulphate electrolyte (Na_2_SO_4_), using a CHI-660E electrochemical workstation (CHI-660E, CH Instruments, Austin, TX, USA).

### 2.4. Photocatalytic Degradation Experiments

A series of photocatalytic degradation experiments of MO (10 mg/L) and MB (10 mg/L) were carried out in aqueous solution under full-spectrum and visible light sources, respectively, to evaluate the photocatalytic activity of the BPTCD/g-C_3_N_4_ heterostructures under these experimental conditions. The absorbance of the degradation products was measured and analysed by UV-Vis spectrophotometer (TU-1900, Beijing, China) to investigate the photocatalytic activity of the heterojunction. The full-spectrum light source for the degradation experiments was a 300 W xenon lamp (equipped with a 390 nm cut-off filter as a visible light source). Specifically, 100 mg of photocatalyst and 100 mL of contaminant solution were placed in a light-avoiding environment and magnetically stirred at 500 rpm for 30 min to ensure that the system reached adsorption-desorption dynamic equilibrium. The mixed solution was transferred to 20 cm directly below the xenon light source, and the full-spectrum or visible light irradiation was turned on; during the photocatalytic process, 1.5 mL of the reaction solution was withdrawn every 10 min to track the decay pattern of pollutant concentration with time. Parallel control experiments were set up for the photodegradation of MO and MB without BPTCD/g-C_3_N_4_ under the same experimental conditions as described above and the photocatalytic degradation of BPTCD and g-C_3_N_4_, respectively, to exclude the interference of non-catalytic factors. In addition, total organic carbon removal rates were confirmed by TOC analysis to guarantee complete mineralisation.

The evaluation of photocatalytic activity can be broadly divided into three categories depending on the application scenario. The first category is dominated by photocatalytic degradation reactions, which generally use organic pollutants as target molecules for degradation. Generally, the reaction kinetics of photocatalytic degradation of organic pollutants conforms to the Langmuir–Hinshelwood quasi-primary kinetic model, and therefore the catalytic activity can be evaluated by the following equation (Equation (1)):

(1) $$Degradation\;efficiency\;(\%)=(\frac{C_{0}-C_{t}}{C_{0}})\times 100\%$$

Here, *C*_0_ represents the initial concentration of the organic pollutant solution, while *C*_0_ denotes the concentration of the organic pollutant solution at time *t*. The fitting was performed using the proposed first-order kinetic model, as shown in Equation (2):

(2) $$ln(\frac{C_{0}}{C_{t}})=kt$$

In this context, *C*_0_ signifies the initial concentration of the organic pollutant solution. *C*_t_ represents the concentration at time *t*, and *k* stands for the apparent rate reaction constant, expressed in min^−1^. Notably, *k* can be derived from the slope of the linear fitting equation.

Quenching experiments were conducted to identify the primary radical species produced during the photocatalytic reaction. In these tests, all scavengers were employed at a uniform concentration of 5 mmol/L; isopropanol (IPA) was used to scavenge hydroxyl radicals (^•^OH), benzoquinone (BQ) for superoxide radicals (^•^O_2_^−^), and EDTA-2Na to trap holes (h^+^_VB_) [31]. Stability assessments comprising five successive cycles were executed on the synthesised samples. Each experiment was executed a minimum of two times, with outcomes presented as average values.

## 3. Results and Discussion

### 3.1. Characterisations

**Figure 1:**
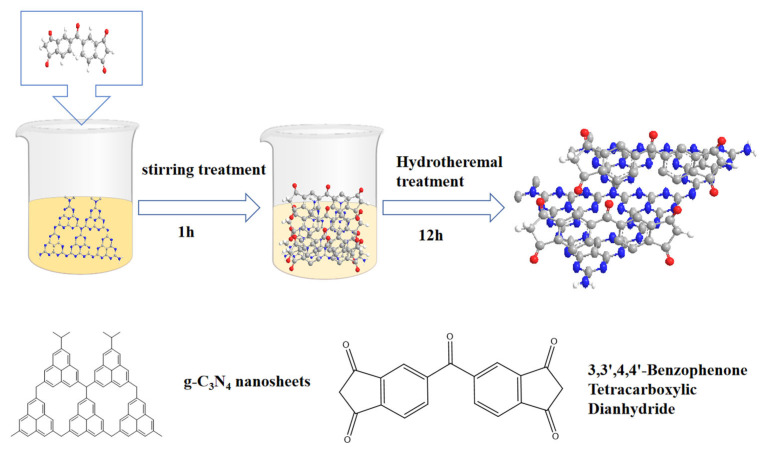
BPTCD/g-C_3_N_4_ heterojunction preparation process.

As shown in Figure 1, this is a schematic diagram of the hydrothermal preparation process for the BPTCD/g-C_3_N_4_ heterojunction. Preparation of g-C_3_N_4_ suspension by the stirring method. In the BPTCD molecule, the carbonyl group is attached to the aromatic ring, and the hydrophobicity of the aromatic ring counteracts the hydrophilicity of the carbonyl group to some extent, rendering BPTCD insoluble in water at room temperature. Since both g-C_3_N_4_ and BPTCD possess aromatic ring structures, their π-π interactions can be significantly enhanced through hydrothermal treatment [32]. Due to the large specific surface area of g-C_3_N_4_ and the π-π bond interaction, BPTCD molecules are adsorbed onto g-C_3_N_4_ via π-π stacking interactions after heating in water.

**Figure 2:**
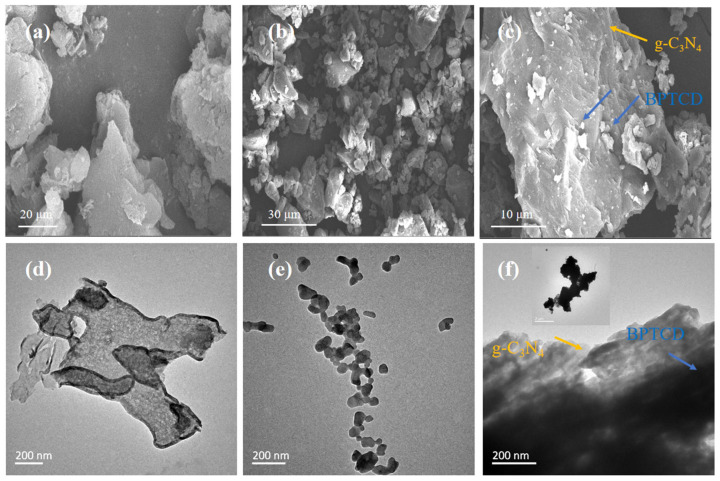
SEM and TEM images of g-C_3_N_4_ (**a**,**d**), SEM and BTDA (**b**,**e**), and SEM and TEM images of BTDA/g-C_3_N_4_ heterojunction photocatalyst (**c**,**f**).

The microscopic morphology and structural features of g-C_3_N_4_, BPTCD, and BPTCD/g-C_3_N_4_ composites were systematically characterised by scanning electron microscopy (SEM) and transmission electron microscopy (TEM). As shown in Figure 2a, the g-C_3_N_4_ sample displays a typical two-dimensional lamellar structure. In Figure 2b, BPTCD has an irregular lumpy particle structure. Figure 2c shows that the obvious morphological features of BPTCD irregular particles and porous layered g-C_3_N_4_ are clearly present on the surface of the BPTCD/g-C_3_N_4_ composites, indicating that BPTCD formed a tight bond with g-C_3_N_4_. Transmission electron microscopy images (Figure 2d,e) further confirmed the two-dimensional lamellar structure of g-C_3_N_4_ and the granular structure of BPTCD. In addition, Figure 2f shows that BPTCD is uniformly distributed on the surface of layered g-C_3_N_4_ through π-π interactions, so the BPTCD/g-C_3_N_4_ heterojunction system is formed. The above results indicate that the basic morphological features of BPTCD and g-C_3_N_4_ were not damaged during the synthesis process, and this unique conformation creates favourable conditions for its enhanced photocatalytic performance [33].

**Figure 3:**
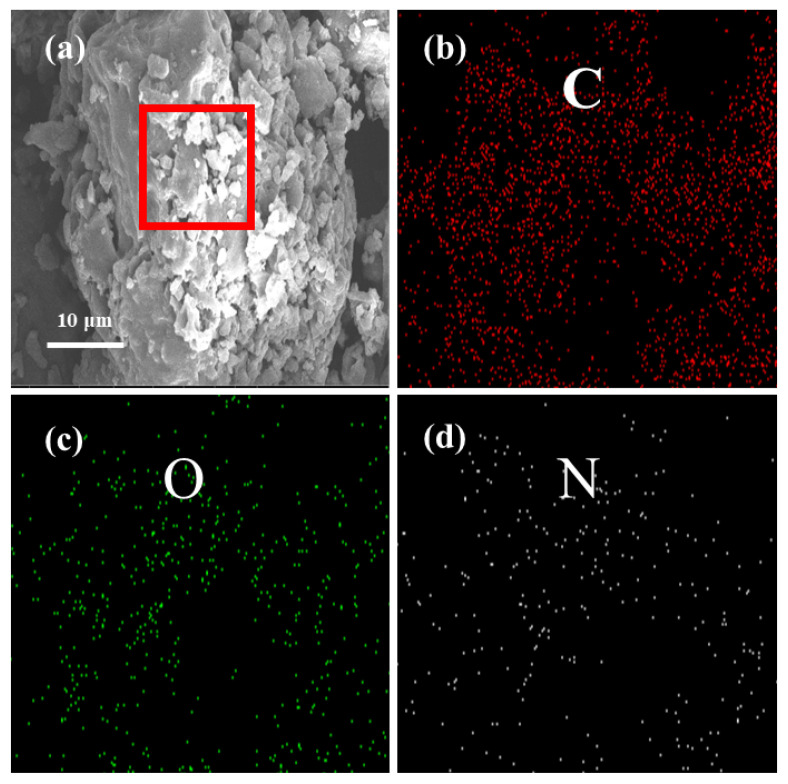
SEM images of BPTCD/g-C_3_N_4_ (**a**) and EDS of corresponding labelled regions (**b**–**d**).

The SEM images of BPTCD/g-C_3_N_4_ and their corresponding elemental distribution maps are demonstrated as shown in Figure 3a–d). The elemental distribution results show that three elements (carbon, nitrogen, and oxygen) are present in the sample, and the results also indicate that these elements are uniformly distributed in BPTCD/g-C_3_N_4_. In addition, Figure S1 provides quantitative information on carbon (C), nitrogen (N), and oxygen (O). The theoretical mass ratio of C to N is 2.2:1, while the actual mass ratio is 2.9:1. The deficiency of N may be due to impurities present in g-C_3_N_4_ during preparation. The higher oxygen peak weight in the spectrum may be attributed to the hydrolysis of BPTCD at high temperatures into tetracarboxylic acid, which significantly increases the oxygen content, and environmental oxygen factors in the scanned sample.

**Figure 4:**
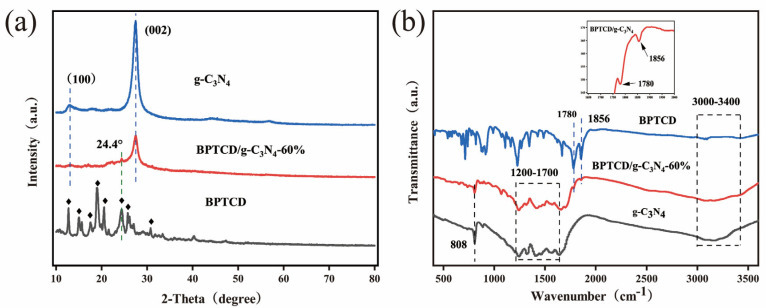
XRD pattern (**a**) and FT-IR spectra of the photocatalysts (**b**).

X-ray diffraction (XRD) was used to characterise the phase composition and crystal structure of the synthesised g-C_3_N_4_, BPTCD, and BPTCD/g-C_3_N_4_ samples. The results are presented in Figure 4a. Focusing on the pore structure of g-C_3_N_4_, two characteristic diffraction peaks (JCPDS number 87-1526) are observed near 13.1° (100 crystal plane) and 27.5° (002 crystal plane) in the X-ray diffraction spectrum, corresponding to the different characteristics of the in-layer long-range ordered aromatic system and the interlayer periodic structural stacking of g-C_3_N_4_ [34,35]. BPTCD exhibits very distinct diffraction peaks at 12.7°, 15.1°, 17.5°, 19.1°, 20.5°, 24.4°, 25.8°, and 30.7°. The diffraction peak of g-C_3_N_4_ at 13.0° disappears after coupling with BPTCD, indicating the presence of strong interactions between the two [31]. As shown in Figure S2, in the BPTCD/g-C_3_N_4_ composites, the diffraction peak of BPTCD at 24.4° is difficult to observe, which is due to the poor crystallinity of BPTCD after hydrothermal heating. The peak at 27.5° is weakened by the BPTCD/g-C_3_N_4_ composites, which may be due to the fact that g-C_3_N_4_ is covered with highly dispersed BPTCD, and this phenomenon can be observed using transmission electron microscopy (TEM) [36,37].

Fourier-transform infrared (FT-IR) analysis was performed on g-C_3_N_4_, BPTCD, and BPTCD/g-C_3_N_4_ samples within 4000–400 cm^−1^ to probe their surface chemistry. As shown in Figure 4b, pristine g-C_3_N_4_ nanosheets exhibit several characteristic peaks between 1200 and 1700 cm^−1^. The peaks at 1242, 1315, 1418, 1571, and 1656 cm^−1^ are attributed to the stretching vibrations of C=N and C-N bonds in CN heterocycles. The strong peak at 808 cm^−1^ corresponds to the vibrational mode of triazine units. Additionally, the peaks in the range of 3400–3000 cm^−1^ are associated with the -NH_2_ groups in the g-C_3_N_4_ nanosheets [38,39]. In the case of BPTCD, the absorption peaks observed at 1780 and 1856 cm^−1^ are attributed to the stretching vibrations of the anhydride (C=O) groups present in the BPTCD structure [23]. No amide (C=O) stretching vibrations were detected at 1660–1667 cm^−1^ in BPTCD/g-C_3_N_4_, indicating that the acid anhydride of BPTCD did not react with the amino group of g-C_3_N_4_, which indirectly proves that non-covalent forces exist between BPTCD and g-C_3_N_4_ [26]. Characteristic signals of g-C_3_N_4_ and BPTCD were detected in the BPTCD/g-C_3_N_4_ photocatalyst, confirming the successful preparation of the composite material. As shown in Figure S3, the FTIR spectra of BPTCD/g-C_3_N_4_ composites with different ratios are similar to those of g-C_3_N_4_, indicating that the addition of BPTCD has not altered its basic structure.

**Figure 5:**
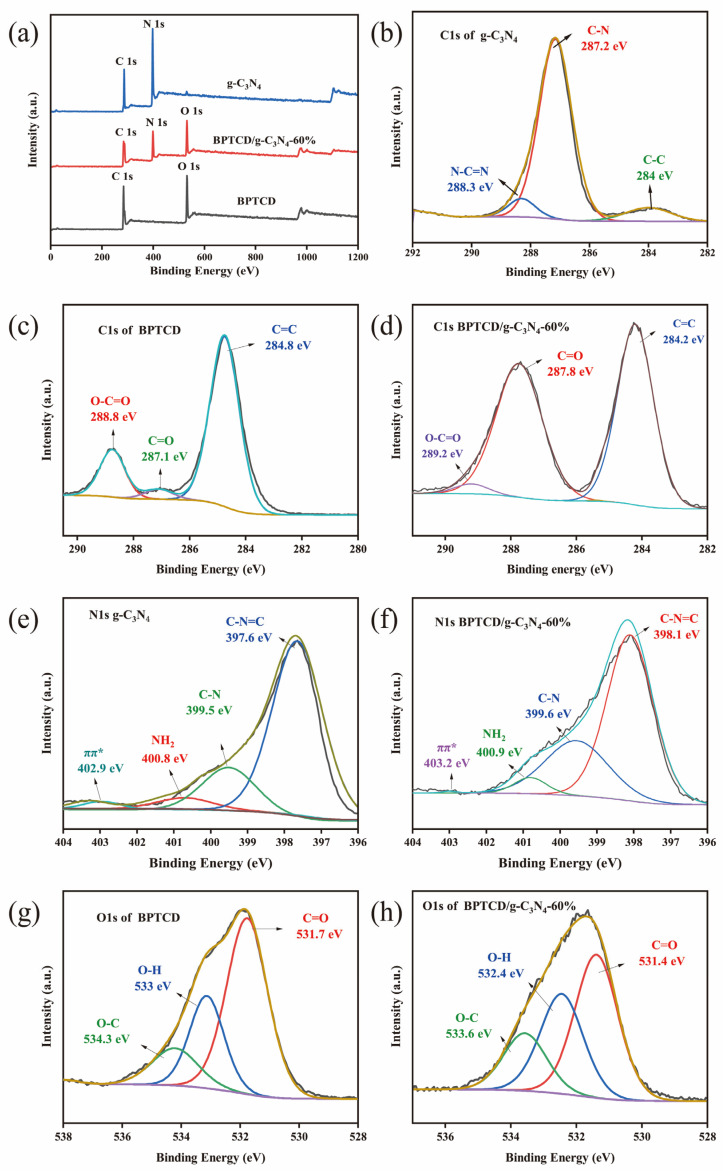
XPS survey spectrum of BPTCD/g-C_3_N_4_-60% heterojunction photocatalysts (**a**); C1s XPS spectrum of g-C_3_N_4_ (**b**), BPTCD (**c**), and BPTCD/g-C_3_N_4_-60% (**d**); N1s XPS spectrum of g-C_3_N_4_ (**e**) and BPTCD/g-C_3_N_4_ (**f**); and O1s XPS spectrum of BPTCD (**g**) and BPTCD/g-C_3_N_4_-60% (**h**).

X-ray photoelectron spectroscopy (XPS) was employed to gain a deeper insight into the chemical characteristics of the samples, with the corresponding findings presented in Figure 5. As illustrated in Figure 5a, the XPS survey spectrum of the BPTCD/g-C_3_N_4_ nanostructure presents distinct C1s, O1s, and N1s peaks, which are in agreement with the EDS analysis results. As depicted in Figure 5b, the deconvoluted C1s XPS spectrum of g-C_3_N_4_ exhibits three distinct peaks positioned at 284 eV, 287.2 eV, and 288.3 eV. These peaks are attributable to the C-C, C-N, and N-C=N functional groups, respectively. As shown in Figure 5c,d, the position of the C=C bond out peak of the BPTCD/g-C_3_N_4_ heterojunction photocatalyst is shifted by 0.6 eV relative to BPTCD towards the lower binding energy, a result that suggests electron transfer and π-π conjugation between g-C_3_N_4_ and BPTCD [40]. As seen in Figure 5e,f, three peaks appear at 398.1 eV, 399.6 eV, 400.9 eV, and 403.1 eV in the spectrum of N1s in BPTCD/g-C_3_N_4_, which belong to the C-N=C, N-C bond, NH_2_ groups, and π-excitation hybridisation of sp^2^ in the triazine ring of g-C_3_N_4_, respectively. It is also noteworthy that an NH_2_ peak appeared on BPTCD/g-C_3_N_4_, suggesting that the NH_2_ groups on g-C_3_N_4_ did not react with BPTCD during the composite process [41,42]. Additionally, as observed in Figure 5g,h, compared to the standalone BPTCD, the O1s spectrum of the BPTCD/g-C_3_N_4_ heterojunction photocatalyst shows a noticeable shift of the C=O bond towards lower energy, indicating that the overall electron transfer is from g-C_3_N_4_ to BPTCD. This is mainly due to the conjugated π-system of g-C_3_N_4_, where electrons migrate from g-C_3_N_4_ to the C=O group of BPTCD. To sum up, the peaks observed in both g-C_3_N_4_ and BPTCD materials are also present in BPTCD/g-C_3_N_4_ heterojunction photocatalysts, confirming the successful integration of g-C_3_N_4_ and BPTCD into BPTCD/g-C_3_N_4_ heterojunction photocatalysts via interfacial interactions.

**Figure 6:**
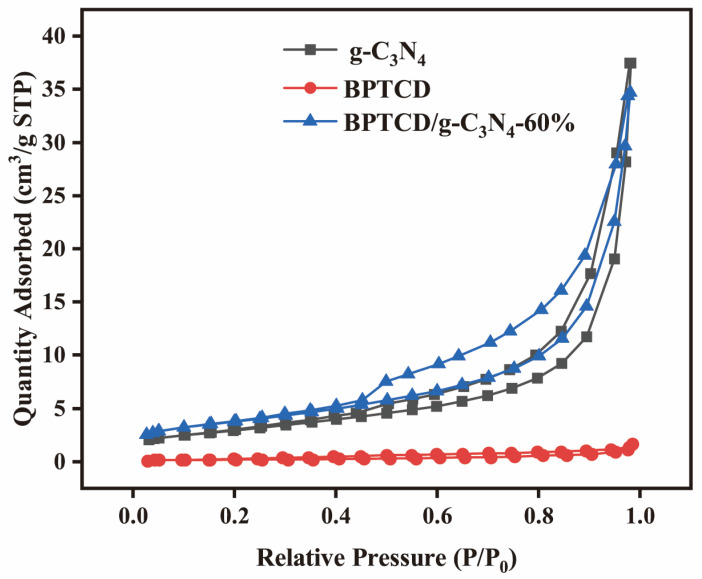
Adsorption-desorption isotherms of g-C_3_N_4_, BPTCD, and BPTCD/g-C_3_N_4_-60%.

The BET method was used to study their specific surface area and pore size distribution. Figure 6 shows the N_2_ adsorption-desorption isotherms of g-C_3_N_4_, BPTCD, and BPTCD/g-C_3_N_4_-60%. These isotherms belong to Type IV, indicating that they have mesoporous structures [43]. The specific surface area, pore volume, and average pore size of g-C_3_N_4_, BPTCD, and BPTCD/g-C_3_N_4_-60% calculated using the BET method are summarised in Table 1. It can be seen that the specific surface area of BPTCD/g-C_3_N_4_-60% (13.518 cm^2^/g) is larger than that of pure g-C_3_N_4_ (11.014 cm^2^/g), thus providing more active sites, facilitating the adsorption of pollutants [44]. The pore volume of BPTCD/g-C_3_N_4_-60% decreased slightly, which may be due to BPTCD being loaded onto g-C_3_N_4_, dividing the large pores on g-C_3_N_4_ into micropores, thereby reducing the pore volume. Furthermore, the pore size of BPTCD/g-C_3_N_4_-60% (1.171 nm) is smaller than that of g-C_3_N_4_ (3.065 nm), potentially because the g-C_3_N_4_ surface is covered by BPTCD and the high temperature and pressure during the hydrothermal process lead to pore wall contraction, resulting in a reduction in pore size.

### 3.2. Photocatalytic Performance of BPTCD/g-C_3_N_4_ in Dye Solutions Under Simulated Sunlight Irradiation

#### 3.2.1. Photocatalytic Degradation of MO

**Figure 7:**
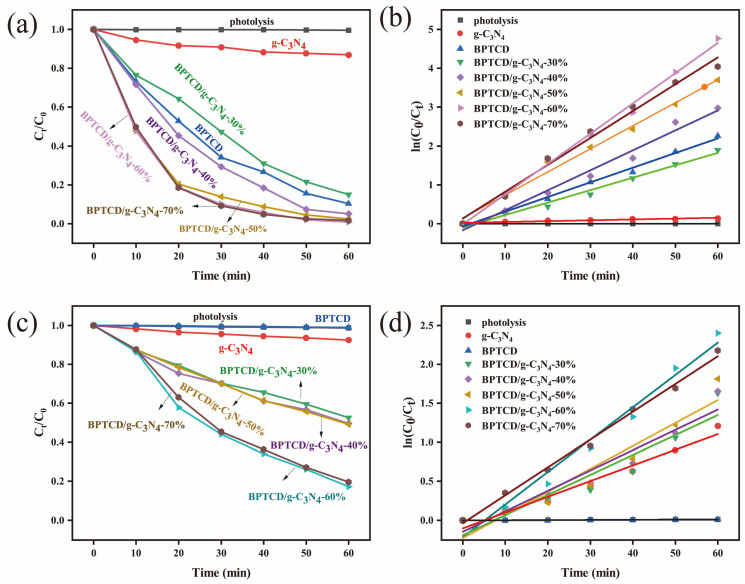
Photocatalytic activity of all synthesised BPTCD/g-C_3_N_4_ materials for MO degradation at full spectrum (**a**), kinetic curve of MO photocatalytic degradation at full spectrum (**b**), photocatalytic activity of all synthesised BPTCD/g-C_3_N_4_ materials for MO degradation under visible light (**c**), and kinetic curve of MO photocatalytic degradation under visible light (**d**).

The degradation performance of photocatalytic materials for methyl orange (MO) was investigated in aqueous systems under full spectrum and visible light. Figure 7a demonstrates the variation in MO concentration under full spectrum, while Figure 7b presents the corresponding distribution of the proposed quasi-primary kinetic constants. This study first examined the photolytic behaviour of the catalyst-free system and found that the degradation rate of MO was only 1% after 60 min irradiation, confirming that it is difficult to effectively decompose this model pollutant by light radiation alone. The system demonstrated a 14.3% MO removal efficiency after the introduction of g-C_3_N_4_ material. The BPTCD/g-C_3_N_4_-30% sample with relatively low BPTCD content (30% mass ratio relative to g-C_3_N_4_) showed 85% photocatalytic degradation of MO after 60 min of full-spectrum light irradiation. It is noteworthy that increasing the mass ratio of BPTCD/g-C_3_N_4_ from 30% to 60% significantly increased the degradation efficiency of MO from 85% to 99.9%. The photocatalytic activity of the BPTCD/g-C_3_N_4_ heterojunction photocatalysts increased gradually with the increase in the mass ratio of BPTCD. This trend indicates that the addition of photoactive BPTCD contributes to the improvement in photocatalytic efficiency. At the same time, BPTCD and g-C_3_N_4_ formed a heterojunction structure through π-π interaction, which effectively enhanced the degradation efficiency of pollutants [45]. It is worth noting that as the mass ratio increases from 60% to 70%, the photocatalytic degradation rate shows an inverse relationship with the BPTCD doping content. This may be due to a reduction in active sites and an elongation of the electron transfer path [46]. The results of photocatalytic kinetic parameters for MO and MB are summarised in Table S1. The quasi-first-order reaction rate constant was measured to be 0.08784 min^−1^ after addition of 60% BPTCD. This value was 2.7 and 30 times higher than that of BPTCD (0.03196 min^−1^) and g-C_3_N_4_ (0.00281 min^−1^), respectively, for a reaction time of 60 min, which indicates that the MO solution has an efficient photocatalytic activity. The BPTCD/g-C_3_N_4_-60% sample exhibited the highest photocatalytic activity. Therefore, the next photoactivity tests were carried out using BPTCD/g-C_3_N_4_-60%. In a comparative study of photocatalytic degradation of MO under visible light, we examined BPTCD/g-C_3_N_4_, BPTCD, and g-C_3_N_4_ with different mass ratios. As depicted in Figure 7c,d, the BPTCD/g-C_3_N_4_-60% photocatalyst accomplished 82.8% MO degradation within 60 min. In comparison, the degradation rate of g-C_3_N_4_ alone under visible light was only 8%, and BPTCD had little photocatalytic effect on the MO solution. These results highlight the excellent photocatalytic performance of the BPTCD/g-C_3_N_4_-60% composite. The corresponding pseudo-primary rate constant of 0.02932 min^−1^ for BPTCD/g-C_3_N_4_ in visible light was 15 times higher than that of g-C_3_N_4_ (0.00126 min^−1^). In summary, the BPTCD/g-C_3_N_4_ heterojunction exhibits excellent photocatalytic activity in both full spectrum and visible light.

**Figure 8:**
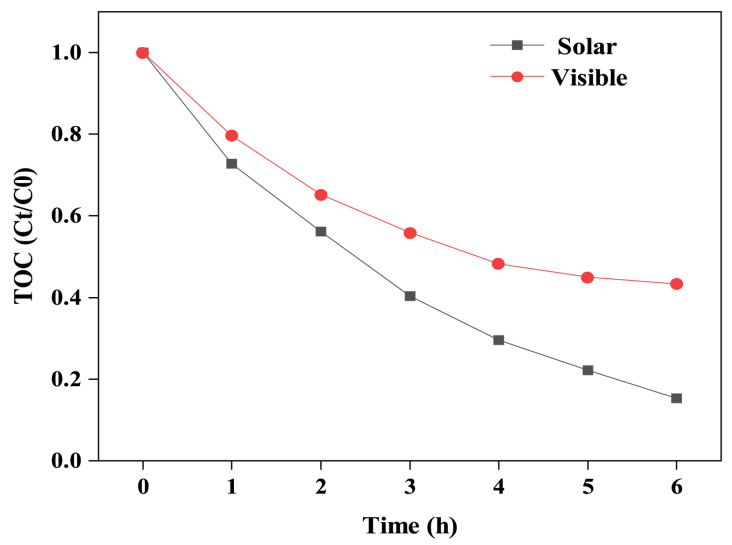
Total organic carbon (TOC) removal efficiency of BPTCD/g-C_3_N_4_-60% heterojunction in the degradation of MO under full spectrum and visible light.

The degree of mineralisation of organic pollutants was determined through TOC testing, further confirming the activity of the BPTCD/g-C_3_N_4_-60% photocatalyst. Although 99% of MO can be degraded within 60 min during the photocatalytic process, it cannot be converted into CO_2_ and H_2_O at the same time [47]. As shown in Figure 8, the mineralisation degree of MO by BPTCD/g-C_3_N_4_-60% under full spectrum and visible light for 6 h was approximately 84.6% and 56.7%, respectively. This is because, during degradation and mineralisation, free radicals preferentially attack the chromophore (-N=N-) to form colourless aromatic amine intermediates. The benzene ring structure of these intermediates is stable, and the sulphate group (-SO_3_H) is difficult to oxidise and remove. This is why the total organic carbon (TOC) remains at a high level [48,49]. However, as time progresses, as shown by the TOC experiment, these intermediate products can be further degraded into CO_2_ and H_2_O.

#### 3.2.2. Effect of pH

**Figure 9:**
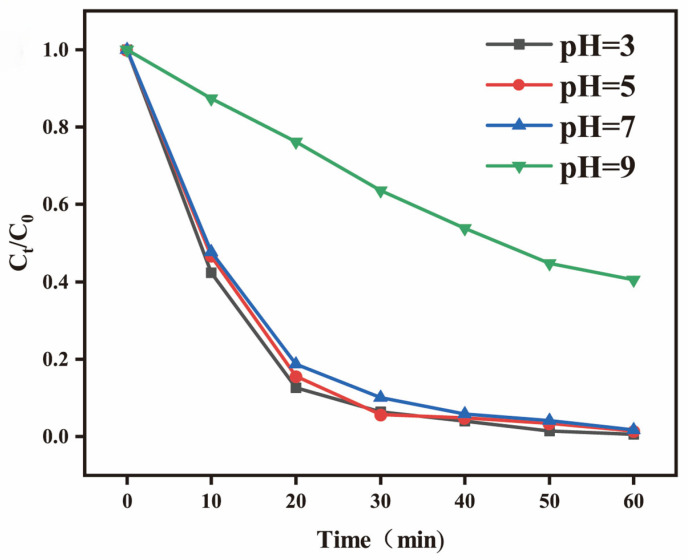
The effect of pH value on the photocatalytic decomposition efficiency of MO under full-spectrum irradiation.

The pH value of the solution is a critical factor influencing the kinetics of photocatalytic reactions. The BPTCD/g-C_3_N_4_ photocatalytic system operates effectively within a pH range of 3 to 9. As can be seen from Figure 9, alkaline conditions are not conducive to the degradation of MO. As the pH value decreased from 9 to 3, the degradation rate of MO was significantly enhanced. At pH = 3, the degradation rate of MO reached 99% within 50 min. This phenomenon can be attributed to the amino protonation and BPTCD interface modification of g-C_3_N_4_. This is consistent with the results obtained in XPS, as the amino group (NH_2_) of g-C_3_N_4_ does not react with BPTCD under acidic conditions. The concentration of h^+^ in the MO solution becomes high, and these groups combine with H^+^ in the solution to form protonated NH^3+^ or NH^2+^, making the surface positively charged. Meanwhile, the electron-withdrawal effect of BPTCD may enhance the protonation ability of g-C_3_N_4_ amino groups or form more surface defective sites under acidic conditions, promoting H^+^ adsorption. In contrast, the surface of BPTCD/g-C_3_N_4_ photocatalysts under an alkaline environment will be negatively charged, while the MO solution under alkaline conditions is also negatively charged. The repulsion between the negative charges is detrimental to the adsorption on the MO solution and the efficiency of photocatalytic degradation [50].

#### 3.2.3. Photocatalytic Active Species and Cyclic Stability

**Figure 10:**
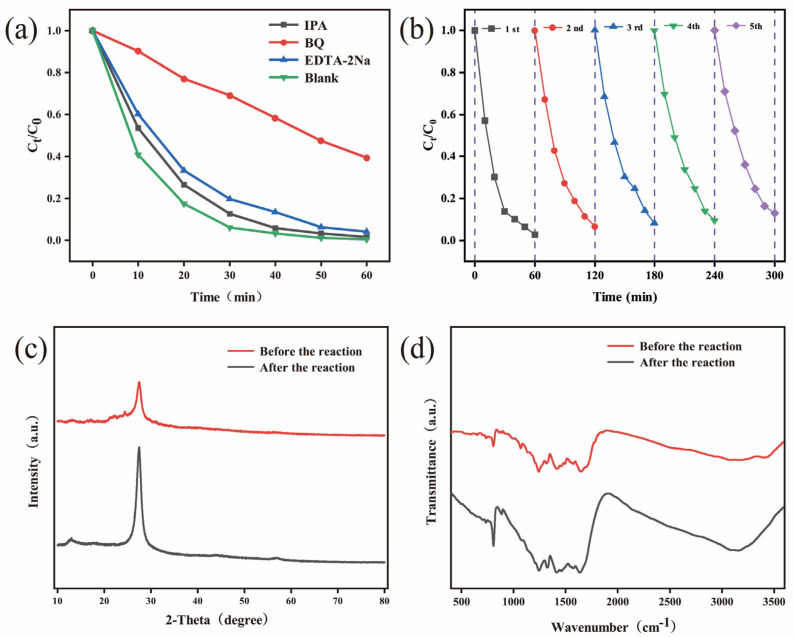
Effect of quencher on MO degradation on BPTCD/g-C_3_N_4_-60% at full spectrum (**a**), reusability of BPTCD/g-C_3_N_4_-60% for MO removal at full spectrum (**b**), and XRD (**c**) and FTIR (**d**) of BPTCD/g-C_3_N_4_-60% photocatalytic heterojunction before and after MO reaction degradation.

The contribution of active species in the photocatalytic degradation of MO induced by BPTCD/g-C_3_N_4_ was analysed through radical scavenging experiments. IPA, BQ, and EDTA-2Na were selected as scavengers for ^•^OH, ^•^O_2_^−^, and h^+^, respectively. Figure 10a shows that the addition of IPA and EDTA-2Na had little effect on the degradation rate, indicating that ^•^OH and h^+^ play a limited role in the reaction. However, the addition of BQ caused the degradation rate to drop sharply to 60.69%, with the rate constant decreasing to 0.0156 min^−1^, indicating that ^•^O_2_^−^ is the key active species in MO degradation. The stability testing of the BPTCD/g-C_3_N_4_-60% sample was conducted via cyclic experiments. As shown in Figure 10b, after five cycles, the photocatalytic activity of the catalyst towards MO remained above 87%, indicating its excellent recyclability [51]. Furthermore, as depicted in Figure 10c,d, there is essentially no discernible difference between the XRD and FTIR spectra of BPTCD/g-C_3_N_4_-60% before and after the reaction, indicating that its structure remains unchanged. This confirms that the heterojunction possesses good stability and reusability.

### 3.3. Degradation of MB by BPTCD/g-C_3_N_4_

**Figure 11:**
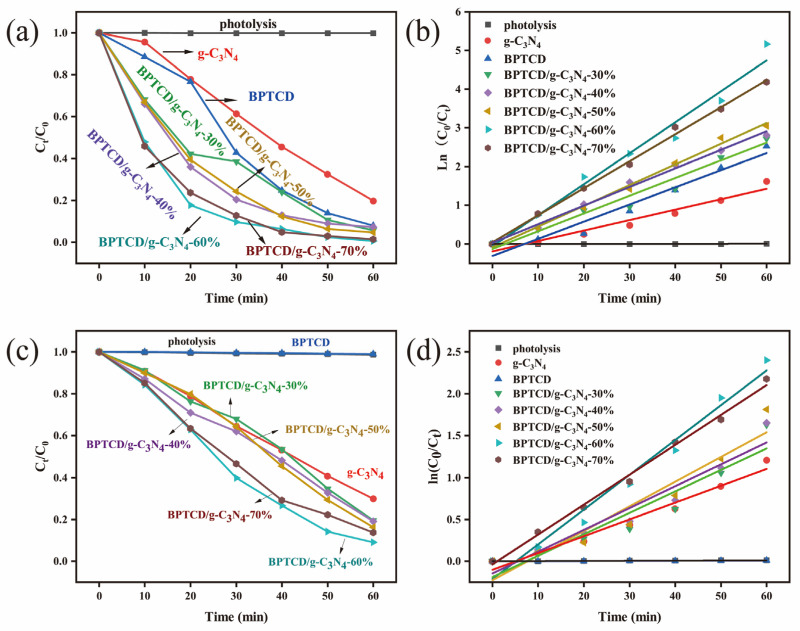
Photocatalytic activity of all synthesised BPTCD/g-C_3_N_4_ materials for MB degradation under the full spectrum (**a**) and MB photocatalytic degradation kinetic curves (**b**). Photocatalytic activity of all synthesised BPTCD/g-C_3_N_4_ materials for MB degradation under visible light (**c**) and MB photocatalytic degradation kinetic curves under visible light (**d**).

In order to expand the application scope of BPTCD/g-C_3_N_4_ photocatalysts, this study also explored their photocatalytic degradation of MB. As shown in Figure 11a, the BPTCD/g-C_3_N_4_-60% photocatalyst achieved 99% MB degradation in 60 min at full spectrum. As shown in Figure 11b, the quasi-primary reaction rate constant of BPTCD/g-C_3_N_4_-60% photocatalyst in MB solution was 0.08808 min^−1^, which was 2.96 times higher than that of g-C_3_N_4_ (0.02698 min^−1^) in 60 min. As shown in Figure 11c,d, the BPTCD/g-C_3_N_4_-60% photocatalyst achieved a 90% degradation rate of MB under visible light within 60 min, while the degradation rate was 70% when using g-C_3_N_4_ alone, and BPTCD itself exhibits almost no photocatalytic activity. Under visible light irradiation, the photocatalytic rate constant of BPTCD/g-C_3_N_4_ reached (0.04152 min^−1^), which is approximately 2.1 times that of g-C_3_N_4_ (0.02009 min^−1^). These results highlight the excellent photocatalytic performance of the BPTCD/g-C_3_N_4_-60% composite material in degrading MB under both full-spectrum and visible light irradiation.

In recent years, g-C_3_N_4_ composite photocatalytic materials have been widely investigated for the degradation of organic pollutants. Table S2 demonstrates the relevant photocatalysts reported by researchers in recent years. Compared with other reported photocatalysts, the photocatalysts prepared in this study showed obvious advantages.

### 3.4. Mechanisms of Enhanced Photocatalytic Activity

**Figure 12:**
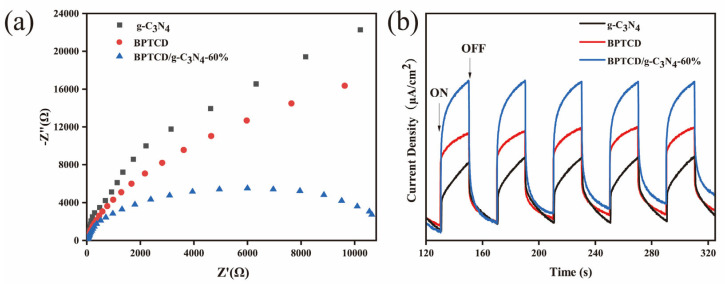
Electrochemical impedance spectroscopy (**a**) and transient photocurrent profiles of g-C_3_N_4_, BPTCD, and BPTCD/g-C_3_N_4_-60% (**b**).

Photoelectrochemical tests were conducted on the materials to gain a deeper understanding of the mechanism behind the enhanced photocatalytic activity of the BPTCD/g-C_3_N_4_ photocatalyst. Electrochemical impedance spectroscopy (EIS) was used to investigate the enhanced interfacial charge transfer. The charge transfer resistance lowers as the arc radius becomes smaller. Figure 12a presents an EIS Nyquist diagram of electrodes composed of g-C_3_N_4_ and BPTCD/g-C_3_N_4_-60%. As observed, the BPTCD/g-C_3_N_4_ photocatalytic exhibits a shorter arc radius compared to that of pure g-C_3_N_4_, indicating a faster interfacial charge transfer. Photoelectrochemical tests were used to probe deeply into the intrinsic mechanisms underlying the significant enhancement of photocatalytic activity in BPTCD/g-C_3_N_4_ photocatalysts. As depicted in Figure 12b, g-C_3_N_4_, BPTCD, and the BPTCD/g-C_3_N_4_-60% composite material all display relatively stable photocurrent densities under sunlight exposure. Notably, the photocurrent density of BPTCD/g-C_3_N_4_-60% is significantly higher than that of samples containing solely g-C_3_N_4_, suggesting that the incorporation of BPTCD, which possesses excellent conductivity, enhances the transport efficiency of photogenerated carriers [52].

**Figure 13:**
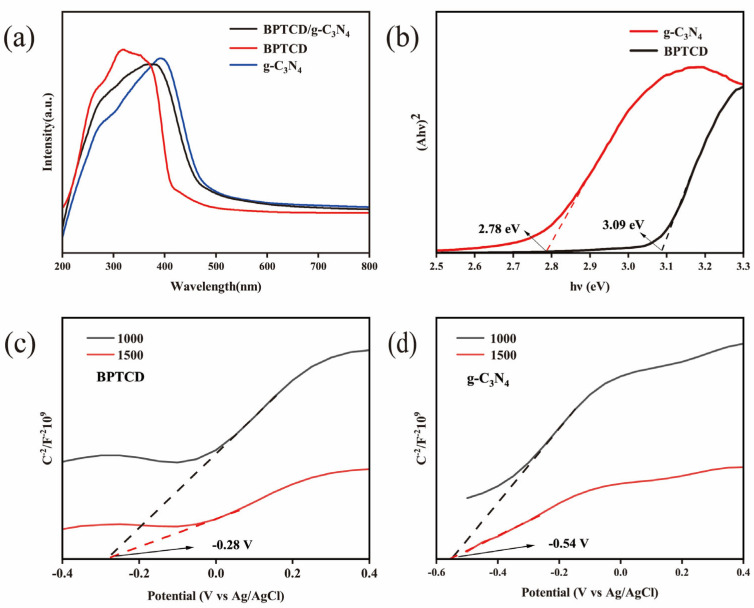
UV-visible diffuse reflectance spectra of BPTCD/g-C_3_N_4_ composites (**a**) and band gap energy profiles of g-C_3_N_4_ and BPTCD (**b**), along with the Mott–Schottky curves of BPTCD and g-C_3_N_4_, respectively (**c**,**d**).

In the assessment of photocatalytic performance, Figure 13a shows the ultraviolet-visible diffuse reflectance spectra (UV-vis DRS) of g-C_3_N_4_, BPTCD, and BPTCD/g-C_3_N_4_ samples. The absorption edges (threshold wavelength, λ) of g-C_3_N_4_ and BPTCD were determined as 460 and 380 nm, respectively. According to the literature, BPTCD has better absorption of ultraviolet light, and its absorption intensity is higher than that of g-C_3_N_4_, which is also the reason why BPTCD exhibits better photocatalytic activity under sunlight [27]. Notably, the absorption edge of the BPTCD/g-C_3_N_4_ photocatalyst extends into the ultraviolet spectrum further than that of g-C_3_N_4_ alone, suggesting that the addition of BPTCD enhances the light absorption properties of g-C_3_N_4_.

These observations indicate that the BPTCD/g-C_3_N_4_ heterojunction system significantly optimises the photocatalytic performance through the synergistic effect of the staggered energy band structure with π-π stacking. Under solar light excitation, the BPTCD/g-C_3_N_4_ heterojunction extends the light absorption range, and its staggered energy band arrangement not only reduces the compound probability of the photogenerated electron-hole pairs, but also the π-π stacking interactions construct an efficient charge transport channel, which synergistically facilitates the directional separation of the carriers and rapid migration [53]. It was further confirmed that the light-trapping ability and charge-transfer property of BPTCD were the dominant factors driving the enhanced activity of the heterojunction photocatalysts. Despite its limited photocatalytic activity, the two-dimensional layered structure of g-C_3_N_4_ can serve as an efficient pollutant enrichment platform to promote the directional migration and spatial segregation of the photogenerated electron-hole pairs of BPTCD through the interfacial adsorption effect, which synergistically enhances the photocatalytic degradation efficiency of the composite system. In order to further elucidate the intrinsic mechanism of photoexcitation and redox reactions, the diffuse reflectance spectroscopy (DRS) data can be converted by the Kubelka–Munk (K-M) function to calculate the optical band gap energy of the semiconductors at the (E_g_). Figure 13b shows a line graph plotting (αhν)^2^ against hν. The band gap widths of pure BPTCD and g-C_3_N_4_ were measured to be 3.09 eV and 2.78 eV, respectively, by Equation (3). In the formula, hν is the incident photon energy (h is Planck’s constant, and ν is the optical frequency), α is the absorption coefficient, A is the proportionality constant related to the material, and n is the index characterising the type of electron jump (the direct band gap is taken as n = 1/2 for direct band gap and n = 2 for indirect band gap). Based on the Mott–Schottky curve analyses (Figure 13c,d), both exhibit typical n-type semiconductor properties (positive slope characteristics). The flat band potentials with respect to the Ag/AgCl reference electrode were further obtained by slope fitting and were −0.45 V and −0.54 V for BPTCD and g-C_3_N_4_, respectively. After conversion to the Normal Hydrogen Electrode (NHE) system according to Equation (4), the flat-band potentials were corrected to be −1.06 V and −1.15 V, respectively [33]. According to Equation (4), the flat-band potentials of the Normal Hydrogen Electrode were found to be −1.06 V and −1.15 V, respectively. By using Equation (5), the position of the edge of the valence band (E_VB_) can be calculated as follows:

(3) $$\left(\alpha h\nu \right)={A\;(h\nu -Eg)\;}^{n/2}$$

(4) $$E_{NHE}=E_{Ag/AgCl}+0.0591\;*\;pH+0.1976$$

(5) $$E_{VB}=E_{CB}+E_{g}$$

Based on the above energy band structure analysis, the valence band edge (E_VB_) of BPTCD is localised at 2.03 V (vs. NHE), while the E_VB_ of g-C_3_N_4_ is 1.63 V (vs. NHE). Under photoexcitation, electrons migrate from the conduction band of BPTCD to that of g-C_3_N_4_, which is attributed to the lower conduction band of g-C_3_N_4_ compared to that of BPTCD. Thermodynamic analyses further revealed that the BPTCD conduction potential was significantly more negative than the reduction potential of O_2_/^•^O_2_^−^ (−0.33 V vs. NHE), indicating that the conduction electrons could effectively reduce adsorbed oxygen to generate superoxide radicals (^•^O_2_^−^) under light conditions, and this inference is in high agreement with the results of free radical trapping experiments [54]. Furthermore, since the conduction band of g-C_3_N_4_ is lower than that of BPTCD, the transfer of photogenerated electrons from g-C_3_N_4_ to BPTCD is demonstrated. The significant differences in valence band and conduction band energy levels between the two phases of the BPTCD/g-C_3_N_4_ heterojunction facilitate the separation and migration of photogenerated carriers, thereby enhancing photocatalytic activity.

**Figure 14:**
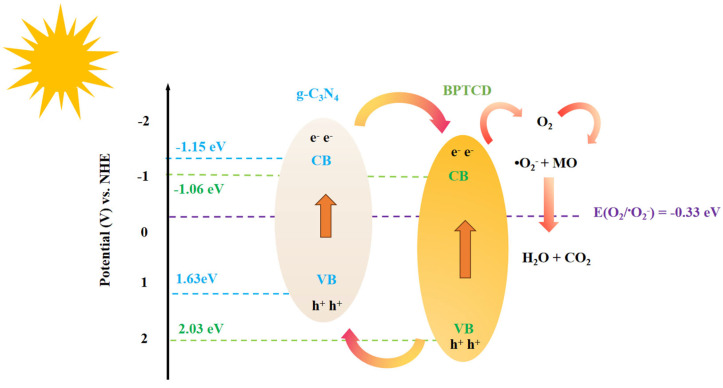
The mechanism of BPTCD/g-C_3_N_4_ heterojunction photocatalyst for MO degradation.

Based on the above characterisation results and discussion, it can be inferred that the BPTCD/g-C_3_N_4_ heterojunction photocatalysts follow a typical type II charge transfer mechanism (shown in Figure 14). When BPTCD/g-C_3_N_4_ was irradiated by sunlight (UV-visible light), photogenerated electrons and holes appeared in the conduction band (CB) and valence band (VB) of the photocatalyst, respectively. In the type II heterojunction formed by the composite of BPTCD and g-C_3_N_4_, there is a close π-π stacking interaction between the two. This π-π stacking interaction enables electrons to form a fast and orderly transport channel between the two. When light irradiates the composite material, photogenerated electrons are preferentially transferred along the channel from the conduction band of g-C_3_N_4_ to the conduction band of BPTCD, whereas the photogenerated holes are efficiently separated at the heterojunction interface and enriched in the valence band region of g-C_3_N_4_. In the heterojunction structure, since the potential of the BPTCD conduction band is more negative than that of O_2_/^•^O_2_^−^, the electrons in the BPTCD conduction band participate in the reduction reaction to capture and reduce O_2_ to ^•^O_2_^−^, which can degrade the MO dye molecules into CO_2_ and H_2_O. The holes in the valence band of g-C_3_N_4_ are involved in the oxidation reaction and can degrade the dyes directly. This effective separation and utilisation of electrons and holes makes the composites significantly enhanced in photocatalytic activity. In visible light photocatalytic systems, although BPTCD itself cannot absorb visible light, it plays an important role as an “electron transfer agent” and “storage reservoir” in heterojunction structures. By receiving electrons from g-C_3_N_4_, BPTCD actively participates in photocatalytic reactions. This mechanism enhances the ability of the entire composite material to utilise visible light energy, thereby significantly improving its responsiveness to visible light.

## 4. Conclusions

In conclusion, we successfully introduced BPTCD into the g-C_3_N_4_ photocatalytic system to optimise the interface, and BPTCD as a charge mediator helps to improve the transfer of photoexcited charges in the interface. The π-π stacking between BPTCD and g-C_3_N_4_ and the staggered energy bands contribute to the separation and migration of photogenerated carriers. The BPTCD/g-C_3_N_4_ heterojunction photocatalysts overcame, to some extent, both the lack of photocatalytic activity of BPTCD under visible light and the inefficiency of g-C_3_N_4_ under visible light. The BPTCD/g-C_3_N_4_-60% degraded MO with efficiencies of 99.9% and 82.8% in full spectrum and visible light within 60 min. Based on the kinetic analysis of photocatalytic reactions, the degradation rates of the BPTCD/g-C_3_N_4_ photocatalyst under full-spectrum and visible light irradiation were 30 times and 15 times higher than those of the original g-C_3_N_4_, respectively. The excellent photodegradation effect of the BPTCD/g-C_3_N_4_ heterojunction on MO is mainly attributed to its composite heterojunction structure and good π-π stacking effect; ^•^O_2_^−^ is the main active component of the heterojunction photocatalytic degradation of MO. Additionally, BPTCD/g-C_3_N_4_ demonstrated good photocatalytic degradation capabilities for methylene blue (MB). The results of this study not only continue the low cost and environmental advantages of g-C_3_N_4_-based materials but also provide solid technical support for the practical application of dye wastewater photodegradation.

## Figures and Tables

**Table 1:** BET specific surface area, pore size, and pore volume of the prepared samples.

Photocatalysts	S_BET_ (m^2^/g^−1^)	Pore Size (nm)	Pore Volume (cm^3^/g^−1^)
g-C_3_N_4_	11.115	3.065	0.058
BPTCD	2.158	2.174	0.003
BPTCD/g-C_3_N_4_-60%	15.085	1.171	0.054

## Data Availability

The original contributions presented in this study are included in the article/Supplementary Material. Further inquiries can be directed to the corresponding author.

## Supplementary Materials

The following supporting information can be downloaded at: https://www.mdpi.com/article/10.3390/nano15141131/s1, Figure S1. EDX spectrum of BPTCD/g-C_3_N_4_-60%. Figure S2. XRD patterns of all samples of BPTCD/g-C_3_N_4_. Figure S3. FTIR spectra of all samples of BPTCD/g-C_3_N_4_. Table S1. Apparent rate reaction constants k (min^−1^) for various types of catalysts in the photodegradation of MO and MB dyes. Table S2. Comparative study of photocatalytic degradation rates of MO by different types of g-C_3_N_4_ matrix composites (see Refs. [55,56,57,58,59,60,61,62,63]).
