# Supplementary material for: Preparation of Non-Covalent BPTCD/g-C3N4 Heterojunction Photocatalysts and Photodegradation of Organic Dyes Under Solar Irradiation
Source: Nanomaterials (Basel). 2025 Jul 21;15(14):1131. doi: 10.3390/nano15141131 (PMC12298509; doi:10.3390/nano15141131)
Supplement: Supplementary file 1 [file nanomaterials-15-01131-s001.zip › nanomaterials-3661569-supplementary.pdf]

Supplementary materials

Preparation of non-covalent BPTCD/g-C_3N_4 heterojunction photocatalysts and photodegradation of organic dyes under solar irradiation

Xing Wei [1], Gaopeng Jia [2,*], Ru Chen [2] and Yalong Zhang [2]

[1] Faculty of Environmental Science and Engineering, Yancheng Institute of Technology, Yancheng 224051, China; 15396805562@163.com
[2] Institute of Textile and Apparel, Yancheng Institute of Technology, Yancheng 224051, China; cr1986634019@163.com (R.C.); 18662029598@189.cn (Y.Z.)
* Correspondences: 15151005525@sohu.com

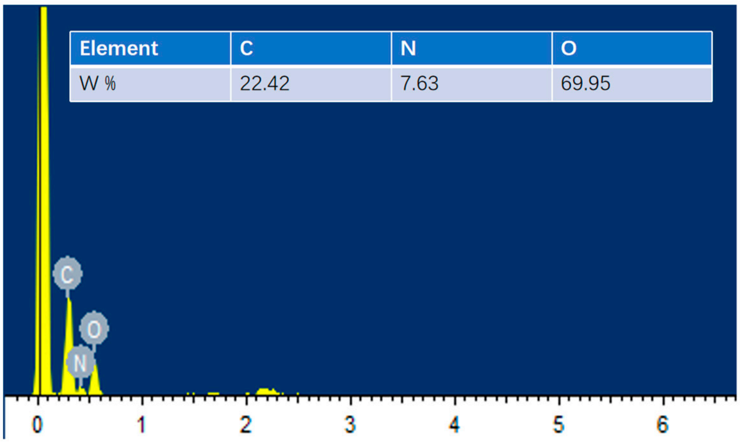

Figure S1. EDX spectrum of BPTCD/g-C_3N_4-60%.

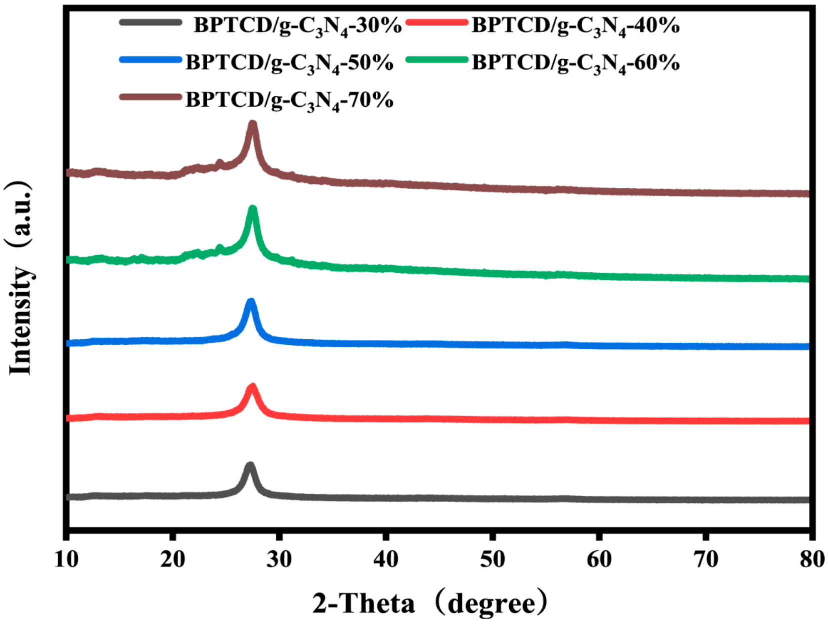

Figure S2. XRD patterns of all samples of BPTCD/g-C_3N_4.

 https://doi.org/10.3390/xxxxx

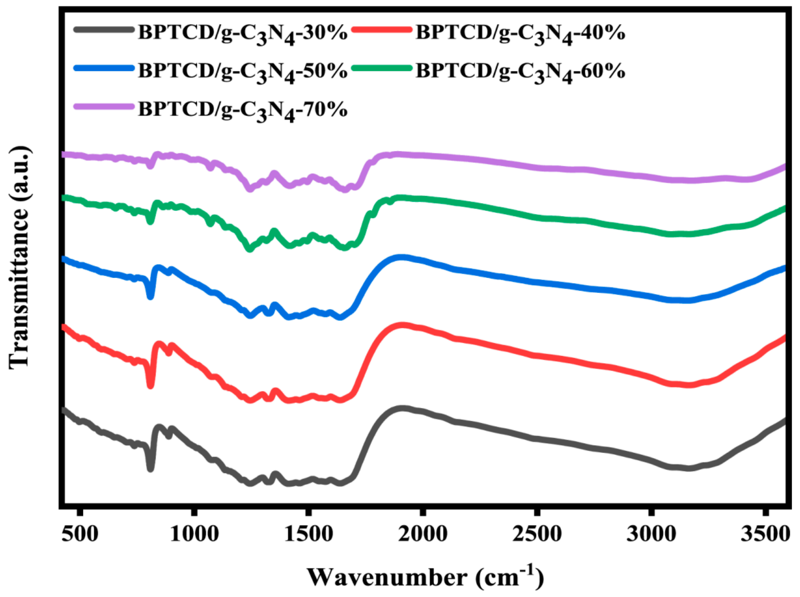

Figure S3. FTIR spectra of all samples of BPTCD/g-C_3N_4.

Table S1. Apparent rate reaction constants k (min^{-1}) for various types of catalysts in the photodegradation of MO and MB dyes.

Samples	Light source	kMO（min^{-1}）	R^2（MO）	kMB（min^{-1}）	R^2（MB）
g-C_3N_4	Solar	0.00281	0.901	0.02698	0.94789
	Visable	0.00126	0.99291	0.02009	0.9706
BPTCD	Solar	0.03196	0.92695	0.04435	0.95435
	Visable	-	-	-	-
BPTCD/g-C_3N_4-30%	Solar	04136	0.9858	0.04582	0.95968
	Visable	0.01031	0.9911	0.02561	0.90094
BPTCD/g-C_3N_4-40%	Solar	0.05133	0.98101	0.04808	0.099329
	Visable	0.01113	0.99446	0.02608	0.93486
BPTCD/g-C_3N_4-50%	Solar	0.05926	0.99002	0.05354	0.99339
	Visable	0.01175	0.9931	0.02943	0.91703
BPTCD/g-C_3N_4-60%	Solar	0.08784	0.99855	0.08008	0.97602
	Visable	0.02932	0.99139	0.04152	0.97561
BPTCD/g-C_3N_4-70%	Solar	0.06905	0.98806	0.06981	0.99648
	Visable	0.02728	0.99674	0.03567	0.99466

Table S2. Comparative study of photocatalytic degradation rates of MO by different types of g-C_3N_4 matrix composites.

photocatalyst	Light source type	Catalyst dosage(mg)	Initial concentration of dye(mg/L)	rate constant(min^{-1})	Reference
Ag/$NaNbO_3$/g-C_3N_4	Solar	100	20	0.03781	1
AgI/g-C_3N_4	Solar	100	5	0.0231	2
g-C_3N_4@V_2C	Visable	100	10	0.02475	3
Ag_3PO_4/Ag/g-C_3N_4	Solar	100	5	0.04126	4
o-GQDs/g-C_3N_4	Solar	100	10	0.0092	5
WO_3/g-C_3N_4	Visible	100	10	0.0213	6
g-C_3N_4/Ag/P_3HT	Visible	60	10	0.0057	7
g-C_3N_4/Bi_2WO_6	Visible	200	10	0.0108	8
CZCM	Visible	50	10	0.0088	9

BPTCD/g-C_3N_4-60%	Visible	100	10	0.02932	This work
BPTCD/g-C_3N_4-60%	Solar	100	10	0.08784	This work

References

1. S.Y.Huang, F. Y. T. Xiong, M. X. Yu, Y. Zhou, J. W. Xu, and J. J. Liu, Synthesis of Ag-loaded $NaNbO_3$/g-C_3N_4 heterojunction for enhanced photocatalytic degradation of methyl orange. Materials Science in Semiconductor Processing. 192, **(2025)**. https://doi.org/10.1016/j.mssp.2025.109401.
2. Y.Orooji, M. Ghanbari, O. Amiri, and M. Salavati-Niasari, Facile fabrication of silver iodide/graphitic carbon nitride nanocomposites by notable photo-catalytic performance through sunlight and antimicrobial activity. Journal of Hazardous Materials. 389, **(2020)** https://doi.org/10.1016/j.jhazmat.2020.122079.
3. R.Z.Xu, G. Y. Wei, Z. M. Xie, S. J. Diao, J. F. Wen, T. Tang, L. Jiang, M. Li, and G. H. Hu, V_2C MXene-modified g-C_3N_4 for enhanced visible-light photocatalytic activity. Journal of Alloys and Compounds. 970, **(2024)**. https://doi.org/10.1016/j.jallcom.2023.172656.
4. Q.. W. Liu, Y. Meng, Q. M. Liu, M. Xu, Y. H.Hu, and S. K. Chen, Synthesis of Ag_3PO_4/Ag/g-C_3N_4 Composite for Enhanced Photocatalytic Degradation of Methyl Orange. Molecules. 28, **(2023)**. https://doi.org/10.3390/molecules28166082.
5. J.HHuang, X. B. Zhang, H. Y. Song, C. X. Chen, F. Q. Han, and C. C. Wen, Protonated graphitic carbon nitride coated metal-organic frameworks with enhanced visible-light photocatalytic activity for contaminants degradation. Applied Surface Science. 441, 85-98 **(2018)**. https://doi.org/10.1016/j.apsusc.2018.02.027.
6. H.Yan., Z. W. Zhu, Y. M. Long, and W. F. Li, Single-source-precursor-assisted synthesis of porous WO_3/g-C_3N_4 with enhanced photocatalytic property. Colloids and Surfaces a-Physicochemical and Engineering Aspects. 582, **(2019)**. https://doi.org/10.1016/j.apsusc.2018.02.027.
7. F.Liu, T. P. Nguyen, Q. Wang, F. Massuyeau, Y. Dan, and L. Jiang, Construction of -scheme-g-C_3N_4/Ag/P_3HT heterojunction for enhanced visible-light photocatalytic degradation of tetracycline (TC) and methyl orange (MO). Applied Surface Science. 496, **(2019)**. https://doi.org/10.1016/j.apsusc.2019.143653.
8. X.M. Gao, R. Zhang, Y. Y. Shang, J. Fei, and F. Fu, Synergism of 3D g-C_3N_4 decorated Bi_2WO_6 microspheres with efficient visible light catalytic activity. Journal of Physics and Chemistry of Solids. 119, 19-28 **(2018)**. https://doi.org/10.1016/j.jpcs.2018.03.032.
9. Z.Y. Zhang, Y. S. Sun, Y. L. Wang, Y. Yang, P. P. Wang, L. F. Shi, L. Feng, S. Q. Fang, Q. Liu, L. Y. Ma, S. Peng, and T. H. Wang, Synthesis and photocatalytic activity of g-C_3N_4/ZnO composite microspheres under visible light exposure. Ceramics International. 48, 3293-3302 **(2022)**. https://doi.org/10.1016/j.ceramint.2021.10.104.
